# Academic literacy among the university students in Mexico and Spain: A holistic perspective

**DOI:** 10.3389/fpsyg.2022.1055954

**Published:** 2023-01-16

**Authors:** Isolda Margarita Castillo-Martínez, Cynthia Patricia Cerros Regalado, Leonardo David Glasserman-Morales, María Soledad Ramírez-Montoya

**Affiliations:** ^1^School of Humanities and Education, Tecnologico de Monterrey, Nuevo León, Mexico; ^2^Business School, Tecnologico de Monterrey, Ciudad Juárez, Chihuahua, Mexico; ^3^Institute for the Future of Education, Tecnologico de Monterrey, Nuevo León, Mexico

**Keywords:** academic literacy, higher education, university students, learning strategies, academic writing, educational innovation

## Abstract

**Introduction:**

Currently, young people have access to a large amount of information, so they must have the ability to critically analyze the texts they are exposed to in order to choose those that are useful for their training or research process, as well as to have the necessary skills to interact efficiently with the texts, especially with those specialized documents corresponding to their area of study. In this regard, this article aims to identify how cognitive, emotional, attitudinal, digital and personality aspects influence the development of academic literacy skills in university students.

**Method:**

Research was conducted with the mixed method, in which quantitative instruments were applied and analyzed: a Likert scale questionnaire to measure the perceived level of mastery of academic literacy skills, which was applied to a sample of 595 students from higher education institutions in Mexico and Spain. In addition, a test was applied to identify personality type. For the qualitative part, the case study was chosen and the qualitative instruments applied were interviews with a teacher and a student and a focus group with five students.

**Results:**

The findings identified were as follows: (a) the cognitive aspect of academic literacy is the one with the lowest perceived mastery by students, (b) having a positive attitude favors the development of academic literacy, (c) by knowing the aspects of their personality that can favor the development of academic literacy, students can seek strategies to improve that competency, (d) the emotional part has repercussions in the process of developing the competence of academic literacy, (e) students prefer to interact with texts in digital spaces and therefore must learn to interact critically in virtual environments, (f) Mexican students perceive themselves to have a higher level of mastery of the academic literacy competency than Spanish students.

**Conclusion:**

The literature review and the mixed methods study allowed identifying the relevance of approaching academic literacy in university environments in a holistic manner through the analysis of the influence of cognitive, attitudinal, emotional, digital and personality aspects.

## 1. Introduction

The complex scenario currently faced by university students makes it necessary to reflect on the competencies that students should have as part of their graduate profile. One of the transversal competencies that is considered fundamental is academic literacy, since it can facilitate the learning process to be able to develop competencies such as efficient information search, comprehension of texts in the area of study to which the students belong, and writing texts with academic rigor. The mixed methods approach was chosen for the research to have a broader view of the object of the study. This study aimed to answer the question: How do cognitive, emotional, attitudinal, digital, and personality aspects influence the development of academic literacy competencies in university students?

A literature review was carried out to identify previous studies related to the topic addressed in this research. Two scoping reviews were identified, one with the objective of exploring and describing methods and strategies to promote the development of academic literacy (Klarare et al., 2022) and the other with the objective of completing a characterization of complex thinking in academic literacy and higher education (Suarez-Brito et al., 2022). Five articles were found that addressed interventions, two of them focused on writing (Olivier, 2019; Zashikhina, 2021), one aimed at academic writing in English (Roux et al., 2018), one about a Center for Reading, Writing and Orality (Soares Sito et al., 2019), and one research focusing on a program that was designed in such a way as to associate learning with the production and analysis of texts specific to the students’ field of study (Urzúa-Martínez et al., 2021), and another article dealt with a cross-cutting program of academic literacy (Pardo-Espejo and Villanueva-Roa, 2019). In contrast, an investigation was also identified, the objective of which focuses on recognizing poor black youth in rural communities as knowledge givers and knowledge takers or “epistemic contributors” (Soares Sito, 2018). Technology was also an element present in some of the studies (e.g., Orlando et al., 2018). To provide information on the condition of academic literacy in Mexico and Spain, a review of studies related to academic literacy in those countries was carried out.

For the specific search of studies in Mexico and Spain, the Scopus database was chosen. A total of 12 studies were found in Mexico using the TITLE-ABS-KEY (“academic literacy” AND “Mexico”) search string. Only those studies that involved a higher education level were selected. A study was identified about the subaltern practices of academic literacy, specifically regarding the use of quotation marks, making a comparison between indigenous and non-indigenous students at the higher education level; in this study, it was concluded that the meanings and practices of literacy that constitute unauthorized variation at the micro and macro levels should be a matter of reflection and educational praxis (Perales-Escudero et al., 2022). Another quantitative research identified aims to determine the relationship between teachers’ prosocial personality traits and their research production and achievements. The authors conclude with a list of training areas that can help alleviate the needs identified from the perspective of information, and scientific and academic literacy (Agredo-Machin et al., 2022). A study was identified that aims to analyze how research-academic literacies are constructed in university programs in the area of social sciences, in three public universities in northern, central, and southern Mexico (Boldo et al., 2018). The article by Mein (2012) analyzes the implications of gender-based research and pedagogy for multilingual students who cross linguistic, cultural, and disciplinary boundaries in their academic studies. Morgan and Carey’s (2009) study considered that one of the main challenges to international students’ right of access to higher education is academic literacy in English and proposes that the adoption of open course models in traditional universities, through blended or online learning, can offer benefits to institutions and non-English-speaking students. Using virtual ethnographic interviews and two online questionnaires, students’ perceptions of the impact of blogs on their academic literacy were analyzed in the study developed by Reyes Angona et al. (2013).

Few studies of academic literacy in Spain could be found. Using the search string TITLE-ABS-KEY (“academic literacy” AND Spain) in the Scopus database, only seven articles were found. A study corresponding to high school students was eliminated. An integrative systematic review was identified to analyze the contextual and pedagogical variables associated with the development and evaluation of the Final Degree Project (TFG). This review made it possible to locate Europe as the continent where most research on TFG has been published (51.85%), with Spain in the first place (64.29%). The study concludes that the TFG involves academic literacy and is an indicator of professional development achieved (Trujillo et al., 2022). Another study related to doctoral theses in Spain focused on analyzing why doctoral students omitted the discussion of results in their theses (Hewitt and Lago, 2010). In another research, the data obtained affirmed that there are differences in students’ literacy associated with the writing and reading practices they perform at the university in response to the demands of their professors (Guzmán-Simón and García-Jiménez, 2017). Other studies identified are specifically situated to English language teaching. The results of Mancho-Barés and Arnó-Macià’s (2017) study shed light on practices and expectations related to the pedagogy of the discipline’s disciplinary genres, an area that is at the crossroads between English-medium instruction (EMI) and English for specific purposes (ESP). In Lillis and Curry’s (2006) research, the results indicate that a significant number of mediators, “literacy brokers,” involved in text production influence texts in different and important ways. In contrast, Taillefer’s (2005) study addresses how students abroad, a product of their own academic literacy culture, face the challenge of integrating quickly into a foreign academic literacy community.

As can be seen, the studies conducted on academic literacy in Mexico and Spain focused mainly on specific aspects, but it is important to conduct studies that analyze academic literacy in a broader sense. It was possible to identify that the studies found focus mainly on the cognitive aspect, only one article was located in Mexico related to the personality aspect, and only two articles were found that addressed the digital aspect, one in each country. Therefore, the present study whose objective is to analyze the influence that cognitive, attitudinal, emotional, digital, and personality aspects may have on the development of academic literacy in Mexican and Spanish university students is relevant.

### 1.1. Academic literacy

It is important to start with the definition of academic literacy that governs this research. Academic literacy is the teaching process that may (or may not) be put in place to promote students’ access to the different written cultures of disciplines. Academic literacy is currently closely linked to competencies to be able to evaluate digital content to subsequently produce knowledge (Carlino, 2013; Solimine et al., 2020).

The components that integrate academic literacy competence are as follows. (a) Attitudes toward academic literacy: the development of self-regulation skills, self-evaluation, metacognition, self-efficacy, and self-esteem as a writer and the attitudes of persistence and frustration tolerance are integrated (Errázuriz, 2017); (b) academic literacy knowledge: the production of texts incorporates knowledge of academic discursive genres and their characteristics, the management of the register to be used in each one, and reflection on the writing process, in addition to the application of cohesion mechanisms (Neira and Ferreira, 2011); (c) academic literacy practice: academic literacy practice involves the use of appropriate strategies for reading and producing (writing and speaking) texts that are considered linguistically and technically appropriate within various academic contexts (Papashane and Hlalele, 2014); and (d) problem-solving skills: they allow for building the problem scenario and similarity of situations that are connected to the learning objectives. They enhance critical thinking (Choon et al., 2021).

In addition to knowing the components that make up academic literacy, among which the cognitive aspect is included, it is important to identify the aspects that influence the development of academic literacy; therefore, it is important to address theoretically the emotional, attitudinal, digital, and personality aspects. In this research, the aforementioned aspects are theoretically supported as follows: the digital element is supported by the Digital Competencies Framework for Citizenship (DigComp 2.1), specifically in Competency Area 1: Information and Digital Literacy, which seeks the development of the following competencies: 1.1 navigate, search, and filter information and digital content, 1.2 evaluate information and digital content, and 1.3 manage information and digital content (Carretero et al., 2017). The attitudinal part is based on the theory of planned behavior (TPB). According to TPB, people’s important behaviors are intentional and, although external and personal constraints make it difficult to act, the immediate determinant of the behavior is the person’s intention to person to perform that behavior (Nuttavuthisit and Thøgersen, 2017). Therefore, TPB proposes the following three variables that influence behavioral intentions: attitude, subjective norms, and perceived control. Attitudes reflect the degree of favorability or unfavorability toward a behavior based on the individual’s acquired beliefs. The subjective norm refers to perceptions of social pressure to engage in a certain behavior by the individual. These arise from the importance people place on the beliefs of others relevant to them, thus regulating their behavior and motivation. Finally, perceived behavioral control considers the perceptions of the relative ease or difficulty of performing the behavior (Galleguillos-Cortés et al., 2022). TPB variables can be identified in academic literacy based on the facts that students can show favorable or unfavorable attitudes toward academic writing, subjective norms are reflected in how students perceive that their teachers, mentors, or facilitators consider that they should perform in the process of writing academic texts pertaining to their area of study, and perceived control is related to how students perceive the ease or difficulty in performing in the process of writing the texts they must produce throughout their student career. The emotional aspect is supported by Lazarus (1968), which holds that, in the face of any threatening event, first, a cognitive appraisal occurs; then, an emotional response is triggered, and, finally, a behavior is adopted to cope with the situation. In this case, when students are faced with the challenge of writing documents specific to their disciplinary area and are not familiar with the process, they may perceive the situation as a threat that leads them to experience negative emotions when faced with the challenge, or they may feel motivated by the challenge they are facing. The theory behind the personality aspect is explained in detail in the following section.

### 1.2. Personality in learning

Each person is different with respect to their abilities, strengths, and the way they perceive their environment, which allows their personality type to influence the way they perform in the different areas of their lives. In the educational field, it is not surprising that the personality and learning style of each student will result in success or failure in their academic life, as well as the development of learning skills (Rosas Prado et al., 2019). Personality is detected through people’s behavior, constitutes a general way of behaving in the circumstances of life, and is mainly generated because of genetic inheritance and interaction with the environment and society (Muela et al., 2010). Thus, personality refers to those traits that unite and differentiate us from the rest of the people to achieve a balance. An important theory of personality is the theory of personal constructs. Kelly (1955) emphasized the creative capacity of living things to represent their environment, as opposed to simply reacting to it. These representations are known as constructs, the patterns that we create in our mind and attempt to fit over the realities of the world. Since our constructs do not always fit with reality, we are constantly modifying them as well as trying to increase our repertoire of constructs. Over time, we tested our constructs for the ability to predict what will happen in our lives. With sufficient time and experience, and if we are willing to learn from our mistakes, we can evaluate all of our interpretations of the world in which we live. In terms of academic literacy, the constructs that people can form are derived not only from the interaction with reality and the perception that arises from it but also from the exposure to content in physical media and digital environments that provide information already digested and that reflect the perception of those who design such content, hence the importance of promoting critical thinking in students when choosing and analyzing the information they have access to through multiple platforms and spaces.

### 1.3. The attitude and emotional part of learning

Attitude is made up of three essential components, which are related to (a) the cognitive component, which is based on the ideas and perceptions about the object of the attitude, (b) the affective component, characterized by the feelings that the person has and their intensity (acceptance-rejection), and (c) the behavioral component, given by the response that the subject has, in reaction to the object of the attitude (Carfora et al., 2022). There are experiential referents, which creates predispositions or attitudes that affect the incorporation of the student into the learning process and affect their school achievement or failure (Gómez Barbosa et al., 2021). Attitudes are learned and relatively stable, so they could be more persistent than habits (Andrade-Valles et al., 2018). Another important element is the emotional aspect. Emotions are closely related to the educational process and the inability to regulate emotional processes can significantly impair the student’s academic performance (Instituto Superior de Estudios Psicológicos, 2021). Attitudinal and emotional aspects are important influencing factors in learning and, therefore, can positively or negatively affect the development of academic literacy.

Several studies were identified related to the influence of attitude with learning and specifically with the performance of complex writing tasks as a part of academic literacy. In education, learning theory, and research, there are a number of constructs that relate closely to attitude and attitudinal learning, including learner beliefs, motivation, self-regulation, and self-efficacy (Watson et al., 2018). Self-efficacy is a type of belief that refers to an individual’s judgment of his or her ability to perform the actions necessary to achieve specific goals (Bandura, 1977). Students with high self-efficacy may have higher confidence and, therefore, be better able to manage the cognitive demands of learning by managing their learning environments through strategies such as self-regulated learning strategies (Komarraju and Nadler, 2013). Self-efficacy is particularly important in completing complex writing tasks. In a study exploring self-efficacy in writing, Pajares et al. (2007) found that how students interpret the results of their own past writing performance, such as how successful they believe they were at completing a writing task, can make a key contribution to their sense of self-efficacy. Graham et al. (2018) found that students’ beliefs (i.e., sense of self-efficacy) contributed to 10% of the variance in predicting students’ writing outcomes and the percentage is even higher (16.3%) for students with disabilities. As can be seen, one aspect that is important to consider for the adequate development of academic literacy is self-efficacy.

The emotional aspect and its link to academic literacy development have also been addressed in previous research. To carry out the coordinated mental activities involved in constructing meaning from text, especially when reading complex texts, a reader must be motivated and put forth effort (Cho et al., 2021). A lack of motivation can be detrimental to reading development because motivation determines how students respond to the challenges of accessing complex readings (Dweck, 1999). Some students may find it motivating to tackle complex readings, but others may feel demotivated and their academic literacy development may suffer.

### 1.4. Academic literacy in the digital scenario

Academic literacy is currently developing in a more complex scenario: the digital scenario. The evolution of technology has always had an enormous impact on all fields of human activity and, in this sense, the digital environment in terms of information search and interaction with texts is no exception. The complexity of the digital scenario not only presents difficulties but also has advantages (Selfa Sastre and Falguera Garcia, 2022). Digital literature presents the reader with a set of texts in which the word is not only the goal of reading but also broadens its horizons toward other analog, visual, and sound codes, thus allowing experimentation linked to the technique, software, and digital possibilities surrounding the literary message (Torres and Côrtes, 2021) and the same can occur with scientific texts in digital environments. The interest in the study of academic literacy is related to the recognition that the initial literacy acquired during primary and secondary education is not sufficient, as it does not allow students to successfully face the demands of a given field of knowledge. Upon entering the university context, students encounter specialized knowledge and, therefore, need to develop specific strategies to engage in the different activities of text analysis and production required for learning in the context of higher education (Carlino, 2013). Academic literacy in higher education becomes an essential element to promote, not only from reading but also from other much more complex competencies, all the human potential (Suarez-Brito et al., 2022). Academic literacy is positioned as a competency worth investigating in the context of higher education.

In México, research has been conducted on academic literacy and digital scenarios. With virtual ethnographic interviews and two online questionnaires, an analysis was made of students’ perceptions of the blogs’ influence on their academic literacy, the links and tensions between the two types of writing, and the possible lessons for educational innovation in the area (Reyes Angona et al., 2013). Another research conducted at the Benemérita Universidad Autónoma de Puebla (BUAP) was identified, which focuses on analyzing the possibilities that a virtual environment through a commercial video game presents for academic writing and publication within the framework of university education (Ponce Carrillo and Alarcón Pérez, 2020). The study of Fernández-Cárdenas and Piña-Gómez (2014) presents the development of a resource portal for academic writing, which illustrates a set of actions and processes that constitute the academic writer’s craft as a social practice. This modeling of academic writing is achieved using a sociocultural and a visual semiotic paradigm. These are some of the studies that show how digital resources and platforms can favor the development of academic literacy. It is important to integrate the development of academic literacy into the graduation profile of university students.

As can be seen earlier, the theoretical framework of this research is based on Carlino’s definition (2013), which conceives academic literacy as the way in which students manage to interact with texts from their field of study and from different disciplines (cognitive aspect) and is enriched with the theories that, in previous paragraphs, explain the attitudinal (TPB), emotional (Lazarus’ Cognitive Appraisal Theory), digital (Digital Competencies Framework for Citizenship), and personality aspects (Kelly’s theory of personal constructs). This theoretical approach allows for a holistic view of academic literacy development.

## 2. Method

For the present research, the Mixed Methods approach was used since the information was collected and analyzed by means of quantitative and qualitative instruments to know the perception on the levels of mastery regarding academic literacy competencies. An explanatory sequential design was chosen since the data obtained through quantitative instruments are enriched with the data collected through qualitative instruments. In the quantitative part, the instrument called eResearch&Literacy (Castillo-Martínez and Ramírez-Montoya, 2020) was applied to 595 higher education students from different institutions in Mexico (383) and Spain (212). **The participating institutions were Tecnologico de Monterrey (various campuses), Universidad del Valle de México (various campuses)**, and **Universidad de Sevilla and Universidad de Cantabria**. As the study was part of the NOVUS OpenResearchLab project of the Tecnologico de Monterrey, the research professors who were part of the project team were in charge of providing support for the application of the eResearch&Literacy questionnaire in their institutions. The application of the questionnaire was carried out online using a Google form.

In the qualitative part, the Case Study approach was chosen. Case studies are analyses in detail of single phenomena that are studied holistically by one or more methods. These single phenomena under study—the cases—may be persons, events, decisions, periods, projects, policies, institutions, or other phenomena or systems (Thomas, 2020). For the present research, an invitation was made to undergraduate professors from universities in Mexico and Spain, obtaining a response from a professor at a university in northern Mexico, so that the case study was constituted with a group of 18 first-semester undergraduate students of the course “Business Strategy and Talent.” The course was chosen to carry out the case study because its professor stated in an interview that his students had difficulties in understanding texts in their area of study, in academic writing, in efficiently searching for information, and in citing according to APA standards. An interview was conducted with a student and a teacher of that course, and a focus group was also carried out with five students of the same course. In the quantitative part, it was also applied to the group of the course analyzed by means of a case study the 16Personalities test (Neris Analytics, 2022), to know their personality type, and through the focus group (qualitative part), it was possible to identify which aspects the students consider that can be useful for the development of the academic literacy competency and which ones can be an obstacle. They also shared the strategies that they find suitable for the development of academic literacy competencies. The analysis of the data collected with the Likert-type eResearch&Literacy questionnaire was carried out with Microsoft Excel, and for the analysis of the qualitative data, the Atlas Ti program was used to identify the codes that later gave rise to the series of categories shown in the “Results” section.

The eResearch&Literacy questionnaire was subjected to a validity and reliability process. Criterion validity was determined, which refers to the strength of the relationship between the measures intended to predict the final criterion of interest and the criterion measure itself (Borneman, 2012). The experts showed a high level of agreement on the ratings given to the criteria of clarity, coherence, relevance, and sufficiency shown in the instrument. A high validity was established between the items of the instrument and the content to be evaluated since an average of 90% was obtained according to the scale used with respect to the degree of agreement. The degree of consistency of this instrument is high since, when applying the split-half reliability statistical method, a result of 0.94 was obtained. A detailed description of the validity and reliability process can be found in the article “Experts’ validation of an instrument for self- perception of research skills to develop academic literacy” (Castillo-Martínez and Ramírez-Montoya, 2020). The questionnaire that was answered as a diagnostic tool to identify students’ perception of their level of mastery of research and academic literacy competencies is shown in Table 1. A four-point Likert-type scale was used for the questionnaire, namely, 1 = strongly disagree, 2 = disagree, 3 = agree, and 4 = strongly agree.

**Table 1:** eResearch&Literacy questionnaire.

Indicators	Items
Attitudes towards research	1. I fulfill my responsibilities in research projects with the guidance of my advisor and/or professor.
	2. I attempt to seek feedback from my advisor or research-related subject teacher regarding my progress on research deliverables at least every 2 weeks.
	3. When I fail to fulfill some academic responsibility, I usually evaluate myself to detect my own mistakes.
	4. I ask for directions if I have any doubts regarding my research work.
	5. I take advantage of opportunities to learn something new in the field of research.
Attitudes towards academic literacy	6. I can accept criticism of my writing and improve it as often as necessary.
	7. I am in the habit of reviewing my papers thoroughly when I complete them before submitting them to my advisor or teacher for revision.
Research knowledge	8. I am familiar with quantitative research methods.
	9. I am familiar with qualitative research methods.
	10. I have knowledge of how to formulate a research question.
	11. I know how to conduct database searches to carry out my research.
	12. I have the theoretical and methodological elements for the elaboration of hypotheses.
Academic literacy knowledge	13. I know the elements of the structure of a research report according to the guidelines of my career.
	14. Regarding a research paper, I know the elements of the structure that are handled in my career.
Research practices	15. I can design research instruments consistently in relation to the research method used.
	16. I can select and/or extract from databases information relevant to my research.
	17. I have the ability to organize and systematize information effectively.
	18. I have the skills for data analysis and interpretation.
	19. I know how to use computer programs for data analysis (Example: SPSS, Minitab, Atlas.ti, QSR Nvivo).
Academic literacy practice	20. I correctly apply spelling and grammatical rules in my writings to report the results of a research process.
	21. I prepare research reports following the structure established for my career.
	22. I elaborate research articles following the structure requested by the scientific journals to which I am addressing.
Research problem solving	23. I contribute to the solution of problems in my area of study through participation in research projects.
	24. I can understand what is happening and what is needed to solve the research problems that are presented to me.
	25. In addition to proposing ideas, I execute actions to solve research problems.
	26. I apply creativity and look for innovative solutions when solving research problems.
	27. I tend to critically evaluate the solutions derived from a given research problem.
Problem solving skills	28. If I do not have enough knowledge about the elaboration of a type of text, I am capable of doing research to elaborate it with the appropriate structure.
	29. If I do not understand a text, I look for strategies that make it easier for me to understand it.

### 2.1. Test to identify personality types

The teacher of the “Business Strategy and Talent” course decided to apply the 16Personalities test so that students could learn about their personality types. This resource was used for the present research to identify the relationship between personality type and the development of academic literacy competencies. The test, as its name suggests, yields 16 personality types, which are classified into four groups, namely, Analysts, Diplomats, Sentinels, and Explorers. The personality types are shown in Table 2.

**Table 2:** Test 16 personalities.

Personality types			
**Analysts** Intuitive and Thinking personality types, known for their rationality, impartiality and intellectual excellence.			
Architect	Logician	Commander	Debater
**Diplomats** Intuitive and Feeling personality types, known for their empathy, diplomatic skills, and passionate idealism.			
Advocate	Mediator	Protagonist	Campaigner
**Sentinels** Observant and Judging personality types, known for their practicality and focus on order, security and stability.			
Logistician	Defender	Executive	Consul
**Explorers** Observant and Prospecting personality types, known for their spontaneity, ingenuity and flexibility.			
Virtuoso	Adventurer	Entrepreneur	Entertainer

The personality types we focused on are those that correspond to the five focus group participants. A **Campaigner** is someone with extraverted, intuitive, feeling, and prospecting personality traits. These people tend to embrace big ideas and actions that reflect their sense of hope and goodwill toward others. Their vibrant energy can flow in many directions. A **Debater** is a person with extraverted, intuitive, thinking, and prospecting personality traits. They tend to be bold and creative, deconstructing and rebuilding ideas with great mental agility. They pursue their goals vigorously despite any resistance they might encounter. A **Protagonist** is a person with extraverted, intuitive, feeling, and judging personality traits. These warm, forthright types love helping others, and they tend to have strong ideas and values. They back their perspective with the creative energy to achieve their goals. An **Entrepreneur** is someone with extraverted, observant, thinking, and prospecting personality traits. They tend to be energetic and action-oriented, deftly navigating whatever is in front of them. They love uncovering life’s opportunities, whether socializing with others or in more personal pursuits.

## 3. Results

As already mentioned in the “Method” section, the participants in this study were 595 university students from Mexico (383) and Spain (212) for the application of the eResearch&Literacy instrument but, for the qualitative part, were carried out through a case study, the group analyzed consisted of 18 first-semester undergraduate students from a university in northern Mexico, of whom 5 participated in the discussion group, one participated in an interview, and a professor was also interviewed. In addition, all 18 students were administered the 16Personalities test. The research results emerged from the application and analysis of quantitative and qualitative instruments, as well as their triangulation.

### 3.1. Quantitative instruments

After applying the eResearch&Literacy instrument to 595 higher education students, we obtained the averages for each dimension of the instrument as well as the overall average regarding the perception of research and academic literacy competencies. For the present research, we focused on the part corresponding to academic literacy, as shown in Table 3.

**Table 3:** Analysis by dimensions of the questionnaire eResearch&Literacy.

	Mean	Standard deviation
Learning by being	3.41	0.64
Learning by knowing	2.78	0.74
Learning by doing	2.94	0.84
Learning by solving	3.24	0.60
Global	3.09	0.76

As can be seen in Table 3, the overall average shows a perception regarding their mastery of academic literacy competencies that is at a high level. In the Learning by Knowing and Learning by Doing dimensions, the average is below 75%. In contrast, the Learning by Solving and Learning by Doing dimensions are above 80%.

An analysis was also performed to identify the averages by item according to academic literacy, as shown in Table 4.

**Table 4:** Means by items corresponding to academic literacy.

Number	Item	Mean	Standard deviation
13	I know the elements of the structure of a research report according to my career guidelines.	2.79	0.75
14	Regarding a research paper, I know the elements of the structure that are handled in my career.	2.78	0.73
20	I correctly apply spelling and grammatical rules in my writings to report the results of a research process.	3.40	0.62
21	I prepare research reports following the structure established for my career.	2.91	0.75
22	I prepare research articles following the structure requested by the scientific journals I write for.	2.52	0.87
28	If I do not have enough knowledge about the elaboration of a text type I am able to research to elaborate it with the appropriate structure.	3.21	0.61
29	If I do not understand a text, I look for strategies that will make it easier for me to understand it.	3.27	0.59

It can be seen in Table 4 that participants perceive a higher level of mastery with respect to the correct application of spelling and grammar rules, which is reflected in their research reports. In contrast, they perceive a lower level of mastery with respect to preparing research articles following the structure requested by the journals to which they are addressed. The attitudinal part of the instrument obtained a perception of the high level of mastery, which is reflected in the averages obtained in items 28 and 29, related to the attitude they show when they do not have enough knowledge about the elaboration of a type of text or do not understand a text.

A comparative analysis of the perception of university students in Spain and Mexico is shown in Table 5.

**Table 5:** Comparative analysis between Mexico and Spain.

		Learning by being	Learning by knowing	Learning by doing	Learning by solving	Overall mean
Mexico	Mean	3.37	2.97	2.96	3.14	3.11
	Standard deviation	0.04	0.05	0.11	0.07	
Spain	Mean	3.19	2.59	2.63	2.93	2.84
	Standard deviation	0.07	0.06	0.11	0.06	

Table 5 shows that there is a perception of a higher level of mastery of the academic literacy competency in Mexican students, and when comparing by dimensions, it can be seen that the perception of the level of mastery in all dimensions is higher in Mexican students than in Spanish students. The variation in the responses is similar in the students of both countries.

### 3.2. Qualitative instruments

An interview was conducted with a teacher and other with a student, in addition to a focus group with five students, and important data were obtained regarding their perception of the personality strengths assigned to them according to the 16Personalities test they answered as well as the points that could be an obstacle to the development of these competencies.

#### 3.2.1. Personality type can promote or hinder the development of academic literacy competencies

In the focus group, although the students were chosen randomly, it turned out that the participants possessed different personality types. They shared the strengths of their personality to facilitate the development of academic literacy competencies and the points that could be an obstacle to achieving their development, as shown in Table 6.

**Table 6:** Helpful and hindering points in the development of academic literacy skills.

	Student 1	Student 2	Student 3	Student 4	Student 5
Personality type	Campaigner	Debater	Protagonist	Protagonist	Entrepreneur
Useful points	*I am very good at generating new skills.*	*I got to be an innovator. I think it’s important to be one step ahead of others, because you are researching, you want to know more and more.*	*The strong point in my personality type is that we like to work as a team. I think that if you do research in a team it is a good way to learn more and have more knowledge.*	*I think a strong point of the animator personality is that he is very communicative, so it is easy for him to communicate and get sources of information, also another one could be that he is a person with a lot of imagination and so on. So, I think that could help a lot.*	*I think I would have to work on that because I tend to get distracted very easily.*
Obstacles	*The obstacles are that if I do not find what I want or expected I get disappointed very easily.*	*One obstacle of my personality I think is my patience, because if I do not see results soon, I need more, more, more, more.*	*Also my personality is kind of easily distracted.*	*As they are so changeable, you do not focus on a single point and you want to cover a lot, so it gets complicated in the end.*	*I think it would be a disadvantage, because I was saying that we cannot concentrate easily, follow a routine, when I’m doing research I get distracted by something else, by the ads that are on the page or so.*
Strategies that facilitate academic literacy development	*When you make a mistake they put in there that you made a grammatical mistake in this and that could help me.*	*Reading helps me more, I understand more when I read. It is always easier for me to understand the text*	*Reading, well, the more I read, the better I get at writing and I think that, yes, so does reading.*	*I feel that the feedback they give you to improve your spelling is more useful to me. If they tell you that you made a mistake, I’ll check my work more.*	*I am more auditory, I have to listen to learn. When I have to learn readings regularly I record myself and then I start listening. If I just read it I do not learn.*

#### 3.2.2. Research competencies can support the development of academic literacy competencies

Although the eResearch&Literacy instrument was analyzed only with respect to the academic literacy competencies portion of the instrument, when conducting the interviews with the teacher and student and the focus group, it was reinforced that there is a close relationship between both types of competencies and that it was important to address it also in this study, which aims to provide a holistic view. The teacher’s (PR) view on this point is reflected as follows:

I believe that everything is governed by the curiosity to investigate. I believe that we can start from there so that a person is able to review, investigate, inquire, formulate, etc. I believe that it is a whole, we could not say that this competence is better for literacy, because it is a whole and somehow in research you will have to go through all the phases, even if it is multidisciplinary.

The student interviewed (S6) responded as follows regarding research skills that can foster the development of academic literacy skills:

Look for reliable information and if you have the support of a teacher, ask him/her as well. Generate that criterion of saying this information is useful, this information is not useful, what am I looking for. If I am going to give proposals to McDonalds, I am not going to give them information that they already have, I am going to generate new information, so how to have my own criteria to say what I am looking for to see what I write.

#### 3.2.3. Personality aspects favor the development of research and academic literacy skills

The participants in the focus group were asked about what strengths they considered their personality type to favor the development of their research and academic literacy competencies. In this regard, S4 comments:

I think one strength of the animator personality is that he is very communicative, so it is easy for him to communicate and get sources of information, also another one could be that he is a person with a lot of imagination and so on. So, I think that could help a lot.

Also, S3 shares:

The strong point in the personality type that came out for me is that we really like to work in a team. I think that if you do research in a team it is a good way to learn more and have more knowledge.

For his part, S2 explains, “I was an innovative person. I think it is important to be one step ahead of others, because you are researching, you want to know more and more” and S1 expresses, “The useful ones would be that I am, well, I said that I am very good at generating new skills.”

#### 3.2.4. Personality aspects hinder the development of research and academic literacy skills

In the focus group, students were led to reflect on what they felt were the aspects of their personality type that hindered the development of research and academic literacy skills. S1 expressed that, for her, the obstacle was, “If I do not find what I want or expected I get disappointed very easily.” S2 shares that, “One obstacle of my personality I think is my patience, because if I do not see results soon is to see what is happening, I need more, more, more, more.” S3 and S5 agree that the main obstacle of their personality type is that they are easily distracted. In contrast, S4 comments, “Being so changeable, you do not focus on a single point and you want to cover a lot, so it gets complicated in the end.”

#### 3.2.5. Difficulties of university students with respect to academic writing

For students, academic writing is not a simple process. Sitting down to write a report, a research report, or an essay, among others, is a complex process. S6 states “I consider myself very good in spelling and grammar and in research in general, but what I struggle with the most is to land my ideas, that is, to draw conclusions, that is what I struggle with the most.”

Also, teachers detect the difficulty students have regarding academic writing. PR shares:

There are serious spelling problems that we have to help them clean up, secondly, they kind of go off into their own thoughts and write themselves, trying to use writing as a kind of outlet, which is fine, I mean, it’s part of what we are working on, but sometimes it’s so much the daily type that they put in there, that suddenly they forget about the academic part.

#### 3.2.6. Influence of the emotional side on academic literacy

A question that has been less explored is how the emotional side can influence proper academic writing and thus efficient academic literacy. PR reflects on how this plays out in students and concludes:

I do have my reflection, I am clear about what I am thinking, but then how do I ground that to the concepts we are seeing. That part of aligning mine with the theoretical part because they go more to the emotional or to what I bring and feel, although maybe in some way they do thread it, when I review it in writing it is not seen and that is what they need to integrate.

The emotional side is also reflected in research work that can serve as a basis for academic literacy, as exemplified in S1’s comment: I started reading everything, but I did not know what I was going to use, almost nothing helped, that’s as far as I got. When the student was asked if he had become unmotivated, he responded: Yes, they were moving very fast and I had to study a lot.

#### 3.2.7. Influence of the attitudinal part on academic literacy

Another aspect that can influence how academic literacy is developed is attitude. An important issue that can positively influence academic writing is the attitude to research, is the attitude to be curious, willing, and also open to constructive criticism regarding research reports to improve academic writing skills and is the attitude research has on a better development of academic literacy competencies. PR expresses that attitude even goes beyond the realm of academic literacy, which impacts everything, as she explains below:

I believe that for everything, organizations can check this part, the attitude, the quality of service, assertiveness, all these personal skills that a person already has as personal skills help you to develop the rest. From having to apply a test to a certain group of the population, to be able to reach a certain group of the population you need to know how to negotiate and communicate effectively and be very assertive, be friendly, if you are not like that, they will hardly want to answer you something, even if it is to know the quality of a product or for marketing purposes, etc.

Some students showed a positive attitude toward research competencies that can favor the development of academic literacy, as in the case of S2: “I like to read other people’s research and then be able to implement them, at the moment if I am liking research.” With respect to academic writing, a positive attitude was identified, as shown in S4’s response: “That is why it is good to get a second opinion, or read it after 2 days to see if it makes sense what you are expressing, maybe there are things you understand but others do not.”

#### 3.2.8. Students prefer to interact with texts on digital platforms

Currently, students can access a large number of texts at the click of a button; they are no longer limited to going to a library to consult a limited collection compared to the vast amount of information provided by the web, the main site where they search for their schoolwork or research. PR reinforces this statement when it shares:

I think that more than anything the digital part, the printed part is not much to their liking, with the digital part they respond better, but if you put a video where you integrate the message you want to provide them with, it is better for them. They prefer listening, watching, not so much the reading part. I mean, there are those who, yes, have that ability more developed, it is a taste they have for reading, but in general, let’s say, 80% are more visual than anything else.

Regarding the search for information on Internet sites, they prefer the web since it is more complex for them to access databases of the digital library of their institution. S6 comments, “Well, the most common is to search in Google, in the open one, Scholar I always forget to use it.” PR also agrees, which is reflected in the following comment:

Google (laughs), the whole web, Wikipedia is almost not used, because they also have already demonized it. I feel that for a while there was a very negative message regarding Wikipedia, so they have already identified it as a space where they can find information, but teachers are not going to accept it. More than anything else on the web, there are some very smart people who use Google Scholar, but it is rare, it is very rare who really uses digital library sources.

## 4. Discussion

Adequate academic literacy in higher education requires solid knowledge of academic discursive genres and the structure of scientific texts. Table 3 shows that university students perceive that they have a lower level of mastery precisely in the cognitive aspect, included in the Learning by Knowing dimension of the eResearch&Literacy instrument with a 2.78. This is specifically observed in the items corresponding to this dimension: “I know the elements of the structure of a research report according to my career guidelines” (item 13 with 2.79) and “Regarding a research paper, I know the elements of the structure that are handled in my career” (item 14 with 2.78). As can be seen in the “Research competencies can support the development of academic literacy competencies” section, S5 reflects on the cognitive aspect that it is very useful to be able to turn to a teacher to improve the knowledge one already has to carry out an efficient search for information. According to Neira and Ferreira (2011), the production of texts should incorporate, in addition to knowledge of academic discursive genres, the management of the register in each one, reflection on the writing process, and knowing how to use cohesion mechanisms. It can be observed that the cognitive aspect of academic literacy is the one with the lowest perceived mastery by students. The cognitive aspect, in addition to being based on knowledge of discursive genres, text structure, and writing process, is also related to an efficient search for information, for which the teacher’s or facilitator’s guidance can be very valuable.

The attitudinal part has an impact on learning in general and, therefore, on the development of academic literacy. Table 4 shows that the items corresponding to attitudes are located in the highest averages with respect to the students’ perception of their level of mastery, and these items are related to having an attitude of openness to search for information if one does not have sufficient knowledge about how to write certain types of texts (item 28) and to the willingness to seek strategies that can facilitate the understanding of texts that are difficult to understand (item 29). In the “Influence of the attitudinal part on academic literacy” section, it is identified that attitude even has repercussions in any field, not only school or academic. Attitude can affect the learning process (Gómez Barbosa et al., 2021). Perceived behavioral control considers the perceptions of the relative ease or difficulty of performing the behavior (Galleguillos-Cortés et al., 2022). Students having a positive attitude about seeking strategies to help them improve the aspects of academic writing with which they are having difficulty is already a critical step, as it will enable them to commit to making progress in developing their academic literacy competence.

Knowing their personality type can have the benefit of helping them identify those areas of opportunity that they can transform to achieve better learning and, in this case, better academic literacy development. Table 6 shows that students with diverse personality types participated in the focus group, and all were able to identify useful points and points that could hinder the development of their academic literacy competencies. Regarding the favorable aspects that the students identified, the creative part stands out, as shown in the responses of S4: to have imagination, S5: to be innovative, and S1: to generate new skills, as well as S4: communication skills and S3: teamwork. It was possible to identify that an important obstacle is impatience, which is shown in the responses of students S1 and S2 regarding feeling disappointed when not finding what they are looking for and wanting to see results quickly, respectively. Another unfavorable point is that they are easily distracted, as in the case of S3 and S5. In the educational setting, it is not surprising that each student’s personality and learning style will result in success or failure in their academic life, as well as the development of learning skills (Rosas Prado et al., 2019). The constructs that students have formed throughout their lives also influence learning (Kelly, 1955) and, in this case, the development of academic literacy. By knowing the aspects of their personality that can favor or disfavor the development of their competencies, in this case, academic literacy, students can seek strategies that lead them to modify the constructs that are preventing them from having a positive disposition for learning and thus, they will achieve potentiating their strengths and improve their areas of opportunity in favor of the development of academic literacy.

The emotional part can also influence adequate academic writing. In the “Influence of the emotional side on academic literacy” section, it is reflected that sometimes students get carried away by the emotional part of their writings and do not take care to integrate what they are writing with the theoretical part of the course for which they elaborate their writings. Emotions are closely related to the educational process and the inability to regulate emotional processes that can significantly impair the student’s academic performance (Instituto Superior de Estudios Psicológicos, 2021). The emotional aspect supported by Lazarus (1968) holds that, before adopting a coping behavior, the threatening event is first cognitively appraised and then an emotional response is triggered. In the case of the group of participants in the study, it is not identified that they perceive the act of writing as a challenge that leads them to experience negative emotions; on the contrary, it seems that the writing process can be for them a catharsis, which leads them to an emotional outlet, which is very positive, but can be an important distractor to achieve the objectives of academic writing and the subject for which they are writing the text. The emotional part has repercussions in any situation and context and the educational environment is no exception, the teaching-learning process may be favored if the student has adequate emotional regulation and whether the facilitator can help students find a balance between expressing their emotions and meeting the academic writing goal.

Learning is now largely framed in digital environments, which also impacts how university students interact with texts. The content of the “Students prefer to interact with texts on digital platforms” section shows how students prefer to read in digital environments; unfortunately, they do not have an adequate level of mastery regarding their digital competencies. Upon entering the university context, students encounter specialized knowledge, and therefore, they need to develop specific strategies to engage in the different text analysis and production activities required for learning in the higher education context (Carlino, 2013). As if that were not enough, they must also be prepared to be able to perform an efficient search for information on digital platforms (Carretero et al., 2017) that put a large amount of information within their reach. In this way, academic literacy implies that the student has the knowledge and skills to interact with texts not only physically but also in digital environments. The students need to improve their ability to understand specialized texts at the university level, especially those in his/her field of study.

An important aspect was to establish an analysis between Spanish and Mexican students to know the difference in their perception of their level of mastery of academic literacy. Table 5 shows that the perceived level of mastery is higher for Mexican students than for Spanish students, taking into consideration the overall mean and mean for each dimension. More studies on academic literacy have been carried out in Mexico than in Spain, according to the literature review carried out in the Scopus database and whose explanation is detailed in the “Introduction” section. The search allowed us to identify that the studies in both countries address specific topics that are fundamentally related to the cognitive aspect, which shows the relevance of studying academic literacy considering a vision that also integrates personality, emotional, attitudinal, and digital aspects.

The literature review allowed us to have a notion of the path taken by researchers regarding the development of academic literacy in university students. In the “Introduction” section, two reviews were found, which were a good contribution to learn about research that shared strategies for the development of academic literacy. It was also valuable to identify several articles that showed evidence of interventions, but it could be seen that the focus was on text analysis and production. The literature review confirmed that the present study generates a contribution to the field of the study of academic literacy in the university context since it contemplates aspects that have not received much attention but which, in the theoretical framework and in the “Results” section of this article, show their importance on aspects, including attitudinal, emotional, and personality, that have been analyzed in various studies involving cognitive and digital, giving rise to a holistic view of the development process of academic literacy competence.

## 5. Conclusion

The present study found that, in relation to the cognitive aspect, it is necessary to reinforce the knowledge of the different types of academic discursive genres and the structure of texts from the time students enter the undergraduate program and to facilitate the comprehension of texts in their area of study and even enable them to understand texts from other disciplinary areas, to favor their participation in interdisciplinary projects. It was also identified that the attitude that students show toward learning may depend on the learning environments that teachers provide so that innovative strategies can be motivating to promote academic literacy. The emotional part should also be considered; the school environment can provide resources that promote emotional regulation in students so that they can have an adequate school performance, which can influence the development of competencies, such as academic literacy in this case.

With respect to personality, it was found that it can be of great benefit to generate spaces and facilitate resources so that students can identify their personality type and their way of learning since they will become aware of their strengths and areas for improvement to design strategies that facilitate their teaching-learning process, allow them to improve their skills and competencies, and favor the development of an efficient academic literacy. Another finding was that students largely interact with texts through digital environments, so universities must seek to promote critical and creative thinking so that they can distance themselves from the contents to which they are exposed and can choose those that are truly in line with their research topics, and it is important to adapt the training to the use of the digital library, taking special care that the information is clear and simple. To ensure that students are trained, it may be necessary to establish formal courses as the teacher interviewed suggests, since having students enter training voluntarily has not yielded results.

It was possible to identify that Mexican students have a higher perception regarding their level of mastery of academic literacy competence than Spanish students. Also, through a literature review, it was found that more studies have been conducted on academic literacy in Mexico than in Spain and that, in both countries, the research focuses on specific aspects, largely in relation to the cognitive and digital aspects. Therefore, a study that addresses academic literacy in university students with a holistic approach is relevant.

The design of strategies that contemplate the elements of the cognitive, attitudinal, emotional, digital, and personality aspects will make it possible to provide students with the possibility of favoring their learning process, generating greater motivation, and making it more meaningful. This will have an impact on the development of competencies necessary for today’s complex environment, among them, the academic literacy competency, which is so important in the graduate profile of university students.

For future research, we recommend the design and validation of an instrument to evaluate the cognitive, attitudinal, emotional, digital, and personality aspects to have more information to provide greater support for the design of strategies that favor the development of academic literacy in university students. Strategies could even be designed to be the curricular integrated into the last grade of high school to generate a valuable bridge that allows students to enter with greater knowledge and skills for academic reading and writing, facilitating their progress in their level of mastery in their academic literacy competence.

## Data availability statement

The original contributions presented in the study are included in the article/supplementary material, further inquiries can be directed to the corresponding author.

## Ethics statement

Ethical review and approval was not required for the study on human participants in accordance with the local legislation and institutional requirements. Written informed consent from the (patients/participants or patients/participants legal guardian/next of kin) was not required to participate in this study in accordance with the national legislation and the institutional requirements.

## Author contributions

IC-M was primarily responsible for designing the study, collecting and analyzing data, and drafting the manuscript. CC participated in the methodology and the writing of the manuscript. LG-M and MR-M contributed to the revision and improvement of the manuscript. All authors contributed to the article and approved the submitted version.

## Funding

This publication is a product of the projects “OpenResearchLab: innovation with artificial intelligence and robotics to scale domain levels of reasoning for complexity” (ID Novus N21-207), funded by the Institute for the Future of Education (IFE) and “Challenge-Based Research Funding Program 2022”. Project ID # I005 - IFE001 - C2-T3 – T. Also, academic support from Writing Lab, IFE Tecnologico de Monterrey, México.

## Conflict of interest

The authors declare that the research was conducted in the absence of any commercial or financial relationships that could be construed as a potential conflict of interest.

## Publisher’s note

All claims expressed in this article are solely those of the authors and do not necessarily represent those of their affiliated organizations, or those of the publisher, the editors and the reviewers. Any product that may be evaluated in this article, or claim that may be made by its manufacturer, is not guaranteed or endorsed by the publisher.
